# 

**Published:** 1901-03

**Authors:** 


### THE BRISTOL

### Medico-Chirurgical Journal.

A JOURNAL OF THE MEDICAL SCIENCES FOR THE WEST OF ENGLAND AND SOUTH WALES

*PUBLISHED UNDER THE AUSPICES OF*

*THE BRISTOL MEDICO-CHIRURGICAL SOCIETY.*

EDITOR:

R. SHINGLETON SMITH, M.D., B.Sc.,

WITH WHOM ARE ASSOCIATED

J. MICHELL CLARKE, M.A., M.D., J. LACY FIRTH, M.S., M.D.,
AND JAMES SWAIN, M.S., M.D.

ASSISTANT-EDITOR:

P. WATSON WILLIAMS, M.D.

EDITORIAL SECRETARY:

JAMES TAYLOR.

"Scire est nescire, nisi id me

Scire alius sciret."

LIBRARY

SURGEON GENERAL'S OFFICE

JUN.-18.-1902

180038.

VOL. XIX.

BRISTOL: J. W. ARROWSMITH.
LONDON: J. & A. CHURCHILL, 7 GREAT MARLBOROUGH STREET.
1901.

WI
BR323

J. W. Arrowsmith

Publisher

Bristol

### Bristol Medico-Chirurgical Society.

---

### PAST PRESIDENTS.

1874—75. FREDERICK BRITTAN, M.D., B.A. Dub.

1875—76. ROBERT WILLIAM COE.

1876—77. SAMUEL MARTYN, M.D. Ed.

1877—78. AUGUSTIN PRICHARD, M.D. Berlin.

1878—79. HENRY EDWARD FRIPP, M.D. Würzburg.

1879—80. WILLIAM MICHELL CLARKE.

1880—81. JOSEPH GRIFFITHS SWAYNE, M.D. Lond.

1881—82. EDWARD LONG FOX, M.D. Oxon.

1882—83. JAMES GEORGE DAVEY, M.D. St. And.

1883—84. WILLIAM JOHNSTONE FYFFE, M.D., B.A. Dub.

1884—85. GEORGE FORSTER BURDER, M.D. Aberd.

1885—86. WILLIAM HENRY SPENCER, M.D., M.A. Cantab.

1886—87. FRANCIS POOLE LANSDOWN.

1887—88. LEMUEL MATTHEWS GRIFFITHS.

1888—89. ROBERT SHINGLETON SMITH, M.D., B.Sc. Lond.

1889—90. NELSON CONGREVE DOBSON.

1890—91. SAMUEL HENRY SWAYNE.

1891—92. FRANCIS RICHARDSON CROSS, M.B. Lond.

1892—93. EDWARD MARKHAM SKERRITT, M.D., B.S., B.A. Lond.

1893—94. JAMES GREIG SMITH, M.B., C.M., M.A. Aberd., F.R.S.E.

1894—95. ALFRED JAMES HARRISON, M.B. Lond.

1895—96. ARTHUR WILLIAM PRICHARD.

1896—97. ALFRED EDWARD AUST LAWRENCE, M.D., C.M. Aberd.

1897—98. JOHN EDWARD SHAW, M.B., C.M. Ed.

1898—99. ROBERT ROXBURGH, M.B. Ed.

1899—00. WILLIAM HENRY HARSANT.

1901.

---

### LIST OF SUBSCRIBERS

TO THE

### Bristol Medico-Chirurgical Journal.

---

*Those marked * are Members of the Society.*

| | |
|---|---|
| Ackland, C. H. | Moorland Court, Poole Road, Bournemouth. |
| Ackland, J. McKno | 24 Southernhay, Exeter. |
| *Ackland, W. R. | 5 Rodney Place, Clifton. |
| Adye, W. J. A. | Church House, Bradford, Wilts. |
| Akers, W. Dutton | The Limes, Oldfield Park, Bath. |
| Aldous, George F. | Charlton House, Mannamead, Plymouth |
| Aldridge, Charles, M.D., C.M. Aberd. | Plympton. |
| Alexander, James, M.D., M.Ch., B.A. Dub. | Bishop's Place, Paignton. |
| Alford, H. T. M. | The Hospital, Weston-super-Mare. |
| Ambrose, J., M.B., C.M. Aberd. | 5 Heath Villas, Eastville, Bristol. |
| Aveline, H. T. S. | Somerset Asylum, Taunton. |

| | |
|---|---|
| *Ballance, H. Stanley, M.D. Lond. | 4 Royal Terrace, Weston-super-Mare. |
| *Barclay, W. M. | 21 Oakfield Road, Clifton. |
| Baretti, T. G. L'Enardi | 49 Royal York Crescent, Clifton. |
| *Barker, G. H., M.B., B.Sc. Lond. | 124 Redland Road, Bristol |
| *Baron, Barclay J., M.B., C.M. Ed. | 16 Whiteladies Road, Clifton. |
| *Basset, Walter | 24 Stow Hill, Newport, Mon. |
| Batten, Rayner W., M.D. Lond. | 1 Brunswick Square, Gloucester. |
| Batten, W. Smith | West Hill, Calne. |
| Bayer Co., Ltd. | 20 Booth Street, Mosley Street, Manchester. |
| *Beddoe, John, M.D. Ed., B.A. Lond., F.R.S. | The Chantry, Bradford-on-Avon. |
| Benham, Harry A., M.D., C.M. Aberd. | Asylum, Fishponds, Gloucestershire. |
| Bennett, W. S. | Escot, Penzance. |

### LIST OF SUBSCRIBERS.

| | |
|---|---|
| Bernard, C. ... ... ... ... ... ... ... ... | Fishponds. |
| *Berry, F. C., M.D., B.Ch., B.A. Dub. ... ... | Burnham, Somersetshire. |
| Berry, James, M.B., B.S. Lond. ... ... ... | 21 Wimpole Street, London, W. |
| Biamonti, Sebastiano ... ... ... ... ... ... | 27 Via Balbi, Genoa, Italy. |
| *Blacker, A. E.... ... ... ... ... ... ... ... | Richmond Hill Avenue, Clifton. |
| *Blagg, Arthur F., M.D. Durh.... ... ... ... | 28 Caledonia Place, Clifton. |
| Board, Edmund C.... ... ... ... ... ... ... | 8 Caledonia Place, Clifton. |
| Boyce, James G. ... ... ... ... ... ... ... | The Chipping, Wotton-under-Edge. |
| Boyd, Geo. S. J. ... ... ... ... ... ... ... | 10 Adelaide Terrace, Portishead. |
| Braine-Hartnell, J. C. R.... ... ... ... ... | Cotswold Sanatorium, Cranham, near Stroud. |
| *Brasher, Charles W. J. ... ... ... ... ... | 128 Cotham Brow, Bristol. |
| Briggs, H. Fielden, D.D.S. Mich.... ... ... | 6 West Mall, Clifton. |
| Brimacombe, R. William ... ... ... ... ... | Lyppard Grange, Worcester. |
| *Bristowe, H. C., M.D. Lond. ... ... ... ... | Wrington, Somerset. |
| Brown, G. Arthur ... ... ... ... ... ... | Tredegar. |
| Brown, William, M.D., C.M. Glasg. ... ... | Fishponds, Gloucestershire. |
| Browne, J. Walton, M.D., B.A. Royal Univ. I. | 10 College Square North, Belfast. |
| *Bullen, F. St. John ... ... ... ... ... ... | 12 Pembroke Road, Clifton. |
| *Bunbury, E. G. ... .. ... ... ... ... ... | 10 Eaton Crescent, Clifton. |
| Burroughs, E. F. H. ... ... ... ... ... ... | 3 Elgin Park, Bristol. |
| *Bush, J. Paul ... ... ... ... ... ... ... ... | Vyvyan House, Clifton Park, Clifton. |

| | |
|---|---|
| *Carr, Alexander ... ... ... ... ... ... ... | 150 Wells Road, Bristol. |
| *Carter, T. M.... ... ... ... ... ... ... ... | Victoria Square, Clifton. |
| *Carwardine, Thomas, M.B., M.S. Lond. ... | 16 Victoria Square, Clifton. |
| *Cave, Edward J., M.D. Lond. ... ... ... ... | 20 Circus, Bath. |
| *Chilton, R. H. ... ... ... ... ... ... ... | Tudor House, Cambridge Park, Bristol. |
| Chute, Henry M. ... ... ... ... ... ... ... | King William's Town, S. Africa. |
| *Clarke, John Michell, M.D., M.A. Cantab. | 28 Pembroke Road, Clifton. |
| Clay, Challoner ... ... ... ... ... ... ... | Manor House, Fovant. |
| Coker, T. V. ... ... ... ... ... ... ... ... | 2 Chesterfield Place, Clifton. |
| Collins, H. W. ... ... ... ... ... ... ... | Wrington. |
| *Cook, Henri, M.D., C.M. Aberd. ... ... ... | 45 Stackpool Road, Bristol. |
| Cooke, A. S. ... ... ... ... ... ... ... ... | Badbrook House, Stroud. |
| Cornall, Miss ... ... ... ... ... ... ... ... | 20 Arley Hill, Bristol. |
| *Corrigan, W. J. ... ... ... ... ... ... ... | Cloughmore, Splott Avenue, Cardiff. |
| Cossham, W. R., M.D., C.M. Aberd.... ... ... | Cirencester. |
| Cotton, William, M.B., C.M., M.A. Ed. ... | 231 Gloucester Road, Bristol. |
| *Coulter, R. J., M.B. Dub. ... ... ... ... ... | Glendower House, Clifton Down. |
| Cousins, J. Ward, M.D. Lond.... ... ... ... | Kent Road, Southsea. |
| Cowan, F. S. ... ... ... ... ... ... ... ... | 27 Queen Square, Bath. |
| Craddock, Samuel... ... ... ... ... ... ... | South Lyncombe, Bath. |
| *Cridland, A. B. ... ... ... ... ... ... ... | Eye Hospital, Bristol. |
| *Cross, F. Richardson, M.B. Lond.... ... ... | Worcester House, Clifton. |
| *Crossman, Edward, M.D. Dur.... ... ... ... | Hambrook, Gloucestershire. |
| *Crossman, F. W. ... ... ... ... ... . ... | Hambrook, Gloucestershire. |
| Crouch, C. Percival, M.B. Lond. ... ... ... | 3 Princes Buildings, Weston-super-Mare |
| Culross, James, M.B., C.M., M.A. Glasg. ... | 66 Queen Street, Newton Abbot. |

| | |
|---|---|
| *Dacre, John ... ... ... ... ... ... ... ... | 14 Eaton Crescent, Clifton. |
| Dalby, Augustus W. ... ... ... ... ... ... | Redland House, Frome. |
| *Davies, D. S., M.D. Lond., D.P.H. Cantab. ... | 60 Oakfield Road, Clifton. |
| Davis, Theodore, M.D. Lond. ... ... ... ... | Beachcroft, Clevedon. |
| Davy, Henry, M.D. Lond ... ... ... ... ... | Southernhay House, Exeter. |
| Dening, Edwin ... ... ... ... ... ... ... | Manor House, Stow-on-the-Wold. |

### LIST OF SUBSCRIBERS.

| | |
|---|---|
| Dickie, James Steel, M.D., C.M. Aberd. ... | Boscombe Park, Bournemouth. |
| *Dobson, Nelson C. ... | Avonmore, Sneyd Park. |
| *Dowling, E. A. G. ... | 26 Pembroke Road, Clifton. |
| Down, A. R. ... | Bampton, Devon. |
| Dunbar, Eliza L. Walker, M.D. Zürich ... | 9 Oakfield Road, Clifton. |
| Eadon, W. F. Bailey ... | Hambrook, Gloucestershire. |
| *Eager, Reginald, M.D. Lond. ... | Northwoods, Winterbourne. |
| *Eager, W. R. ... | Northwoods, Winterbourne. |
| Eberle, Emily, M.A., M.B., B.Ch. Dub. ... | 17 Oakfield Road, Clifton. |
| *Edgeworth, F. H., M.B., B.C., B.A. Cantab., B.Sc. Lond. ... | 35 Pembroke Road, Clifton. |
| Elder, William ... | 7 Trelawney Road, Bristol. |
| *Elliott, Christopher, M.D., B.A. Dub. ... | 88 Pembroke Road, Clifton. |
| Elliott, F. Percy, M.B., C.M. Aberd. ... | 113 Grove Road, Walthamstow, London, N.E. |
| Ellis, W. McD., M.D. Brux. ... | Cedar Lodge, Bath. |
| Essex, J. R. ... | Pontypool, Monmouthshire. |
| Evans, T. G. C. ... | Budleigh Salterton, Devon. |
| *Ewens, John ... | 17 Redland Grove, Bristol. |
| Fairbanks, W., M.D., C.M. Ed. ... | Wells, Somersetshire. |
| *Fawcett, E., M.B., C.M. Ed. ... | 141 Redland Road, Bristol. |
| *Fendick, R. George ... | St. Paul's Road, Clifton. |
| Finch, Thomas, M.B., B.A. Cantab. ... | Tamworth, Babbacombe. |
| *Firth, J. L., M.D., B.S. Lond. ... | 20 Richmond Park Road, Clifton. |
| *Fisher, Theodore, M.D. Lond. ... | Harley Lodge, Clifton. |
| *Flemming, A. L. ... | The Asylum, Fishponds. |
| *Flemming, C. E. S. ... | Freshford. |
| Ford, Joseph ... | Wedmore. |
| Forty, D. H. ... | Wotton-under-Edge. |
| Fowler, O. H. ... | Cirencester. |
| *Fox, Bonville B., M.D., M.A. Oxon. ... | Brislington. |
| *Fox, E. Long, M.D. Oxon. ... | 4 Clifton Park, Clifton. |
| Fox, F. F. ... | Yate House, Chipping Sodbury. |
| Fox, John Alfred ... | Tregea House, Morrah Road, Penzance. |
| Fox, T. Colcott, M.B. Lond., B.A. Cantab. ... | 14 Harley Street, London, W. |
| Frazer-Toovey, T. E. ... | The Hospital, Teignmouth. |
| Freeman, J., M.D. Brux. ... | 24 Zetland Road, Bristol. |
| *Fyffe, W. Johnstone, M.D., B.A. Dub. ... | 2 Rodney Place, Clifton. |
| Gamble, C. H. ... | Barnstaple. |
| Garland, E. B., M.B., C.M. Edin. ... | 92 Hampton Road, Redland. |
| Genge, T. T. ... | 21 Whiteladies Road, Bristol. |
| Gent, W. C. ... | 170 Wells Road, Bristol. |
| *Gerrish, D. S. ... | Cambridge House, Whitehall, Bristol. |
| *Gibbs, Alfred N. Godby ... | 52 Whiteladies Road, Clifton. |
| Gidley, G. G. ... | Cullompton, Devon. |
| Goss, T. Biddulph ... | 1 Circus, Bath. |
| Grace, Alfred ... | Chipping Sodbury. |
| Grace, Edward Mills ... | Thornbury, Gloucestershire |

Gratte, C. Brooke ... ... ... ... ... ... 183 Commercial Road, Newport, Mon
*Greer, W. J. ... ... ... ... ... ... ... ... 2 Chepstow Road, Newport, Mon.
Grey, T. C. ... ... ... ... ... ... ... ... Camborne, Cornwall.
*Griffiths, J. S. ... ... ... ... ... ... ... 25 Redland Park, Bristol.
*Griffiths, L. M. ... ... ... ... ... ... ... 11 Pembroke Road, Clifton.
Griffiths, P. Rhys, M.B., B.S. Lond. ... ... 71 Newport Road, Cardiff.
*Groves, E. W. H., M.B., B.Sc. Lond. ... ... Kingswood, Bristol.
Groves, J., M.B., B.A. Lond. ... ... ... ... Carisbrooke.

Hadwen, W. R., M.D. St. And. ... ... ... ... 34 Brunswick Square, Gloucester.
*Hailes, Clements D. G., M.D., C.M. Ed: ... 27 Alma Road, Clifton.
*Haines, A. H. ... ... ... ... ... ... ... ... 8 Cotham Road, Bristol.
Handfield-Jones, Montagu, M.D. Lond. ... 35 Cavendish Square, London, W.
Harper, Joseph ... ... ... ... ... ... ... Bear Street, Barnstaple.
*Harris, H. Elwin, B.A., M.B. Cantab. ... ... 13 Lansdown Place, Clifton.
*Harrison, A. J., M.B. Lond. ... ... ... ... Guthrie Road, Clifton.
Harsant, J. G., M.D., B.S. Lond. ... ... ... Exeter Road, Bournemouth.
*Harsant, W. H. ... ... ... ... ... ... ... Tower House, Clifton.
*Harvey, Alfred, M.B. Lond. ... ... ... ... Westbury-on-Trym.
Hawkins-Ambler, G. A. ... ... ... ... ... 67 Rodney Street, Liverpool.
Hay, M. B. ... ... ... ... ... ... ... ... ... North Devon Infirmary, Barnstaple.
Hayman, Alfred G. ... ... ... ... ... ... ... Hapsford House, near Frome.
*Hayman, C. A., M.D. St. And. ... ... ... ... Kingston Villa, Richmond Hill, Clifton
Heath, Miss ... ... ... ... ... ... ... ... 7 Gwynn Street, Bristol.
*Heaven, John C., D.P.H. Lond. ... ... ... 17 Whiteladies Road, Bristol.
*Herapath, C. K. C. ... ... ... ... ... ... ... Cotham Park, Bristol.
*Hetling, H. E. ... ... ... ... ... ... ... 30 Elliston Road, Bristol.
Hill, Hedley ... ... ... ... ... ... ... ... 1 Redcliff Hill, Bristol.
*Hill, Thomas, M.D. St. And. ... ... ... ... 138 Cromwell Road, Bristol.
*Hill, Walter J. ... ... ... ... ... ... ... ... Edgcumbe Villa, Clevedon.
Hoffman, A. W. W. ... ... ... ... ... ... ... 30 The Parade, Leamington.
Hollings, Edwin T. ... ... ... ... ... ... Corporation Road, Newport, Mon.
Horder, T. Garrett ... ... ... ... ... ... 32 Windsor Place, Cardiff.
Hospital, Bristol General (*Sec.*, W. Thwaites).
Hospital, Taunton and Somerset (*House-Surgeon*, W. Drake).
Howells, William, M.B., C.M. Glasg. ... ... Watton House, Brecon.
Huggard, W. R., M.D., M.Ch., M.A. Roy. Univ. I. ... ... ... ... ... ... ... ... Davos-Platz, Switzerland.
Hunt, A. D. ... ... ... ... ... ... ... ... Millbrook House, Chagford.

Imlay, G. A., M.B., C.M. Aberd. ... ... ... 1 Cotham Park, Bristol.
Ingle, C. Durban ... ... ... ... ... ... ... Somerton, Somerset.
Ives, William R. Y. ... ... ... ... ... ... 6 Portswood Hill, Bevois Hill, Southampton.

Jackson, Mark, M.D. Roy. Univ. I. ... ... ... Bear Street, Barnstaple.
Jacobson, W. H. A., M.B., M.C., M.A. Oxon. 66 Great Cumberland Place, London, W.
Jamison, A., M.B., B.Ch., M.A. Roy. Univ. I. ... 66 Woodstock Road, Belfast.
Jones, Evan ... ... ... ... ... ... ... ... Ty-mawr, Aberdare.
Jones, H. Macnaughton, M.D., M.Ch. Roy. U.I. ... ... ... ... ... ... ... ... ... 131 Harley Street, London, W.
Jones, W. W., M.B., C.M. Ed. ... ... ... ... 47 Wellington Street, Merthyr Tydvil.

*Kemm, F. St. J. ... ... ... ... ... ... ... Worle.
Kendall, Bernard C. ... ... ... ... ... ... Cross Street, Helston, Cornwall.

| | |
|---|---|
| *Kendall, H. W. ... ... ... ... ... ... ... | 1 Burlington Road, Redlan |
| Kennedy, W. P., M.D., B.Ch., B.A. Dub. ... | High Street, Lydney. |
| Kinneir, R. ... ... ... ... ... ... ... ... | The Tower House, Malmesbury. |
| *Kyle, H G., M.B., B.Ch. Oxon.... ... ... ... | Bristol General Hospital. |

| | |
|---|---|
| *Lace, F. ... ... ... ... ... ... ... ... ... ... | 1 Paragon, Bath. |
| Lancaster, E. Le Cronier, B.A., M.B., B.Ch. Oxon. ... ... ... ... ... ... ... ... ... | Winchester House, Swansea. |
| *Lansdown, F. Poole ... ... ... ... ... ... | 19 Whiteladies Road, Clifton. |
| *Lansdown, R. G. P., M.B., B.S. Dur. ... ... | 39 Oakfield Road, Clifton. |
| *Lawrence, A. E. Aust, M.D., C.M. Aberd. ... | 19 Richmond Hill, Clifton. |
| Lawrie, J. Macpherson, M.D., C.M. Glasg.... | Greenhill, Weymouth. |
| Le Soudier, H. ... ... ... ... ... ... ... | 174 et 176 Boulevard St. Germain, Paris. |
| *Lees, E. Leonard, M.D., C.M. Ed. ... ... ... | Chantry Road, Clifton. |
| Leigh, W. W. ... ... ... ... ... ... ... ... | Treharris, Glamorganshire. |
| *Leonard, R. C., M.D. Ed. ... ... ... ... ... | 134 Coronation Road, Bristol. |
| Library, Columbus Medical ... ... ... ... | 103 State Street, Chicago, U.S.A. |
| *Ligertwood, Walter ... ... ... ... ... ... | Bath and Somerset County Asylum, Wells. |
| Linden, H. Cooke ... ... ... ... ... ... ... | Compton Martin, Somerset. |
| Lindsay, James A., M.D., M.Ch., M.A. Roy. U.I. ... ... ... ... ... ... ... ... ... | 37 Victoria Place, Belfast. |
| Lister, Lord, P.R.S. ... ... ... ... ... ... | 12 Park Crescent, London, N.W. |
| *Lowe, T. Pagan ... ... ... ... ... ... ... | 16 Circus, Bath. |
| *Lucas, J. J. S. ... ... ... ... ... ... ... ... | Wells Road, Totterdown. |
| Lucy, Reginald H. ... ... ... ... ... ... | 9 Princess Square, Plymouth. |

| | |
|---|---|
| *McCrea, P. W., M.D., B.Ch., B.A.O., B.A. Dub. ... ... ... ... ... ... ... ... ... | 19 Portland Square, Bristol. |
| MacDonald, P. W., M.D., C.M. Aberd.... ... | County Asylum, Dorchester. |
| McElfatrick, John ... ... ... ... ... ... | Stonycroft Grange, Alderley Edge, Cheshire. |
| *McKee, A. B., M.B., B.Ch. Dub. ... ... ... | Sussex Villa, Sussex Place, Bristol. |
| *Mackie, F. P. ... ... ... .. ... ... ... ... | Fylton Rectory, near Bristol. |
| Mackinnon, Charles, M.B., C.M., M.A. Glasg. | Dyer Street, Cirencester. |
| Maclean, Ewen J., M.D., C.M. Ed.... ... ... | 51 Linden Gardens, London, W |
| Maclean, J. Campbell, M.B., C.M. Ed.... ... | Swindon House, Swindon, Wiltshire. |
| Macready, J. ... ... ... ... ... ... ... ... | 42 Devonshire Street, Portland Place, London, W. |
| McWatters, J. C. ... ... ... ... ... ... | Almondsbury. |
| Marsh, O. E. Bulwer ... ... ... ... ... ... | Parkdale, Newport, Mon. |
| Marshall, Arthur L., M.B., B.A. Cantab. ... | Thornleigh, Upper Clapton, London. |
| *Martin, E. F., M.B. Ed. ... ... ... ... ... | Victoria House, Weston-super-Mare. |
| *Martin, J., M.D. St. And. ... ... ... ... ... | Rosslyn, Clevedon. |
| *Martin, R. C. ... ... ... ... ... ... ... ... | 3 Clifton Vale, Clifton. |
| *Martyn, G. J. King, M.D., B.C., B.A. Cantab. ... ... ... ... ... ... ... ... ... | 12 Gay Street, Bath. |
| Mason, S. B. ... ... ... ... ... ... ... ... | 12 Park Terrace, Pontypool. |
| Mason, William ... ... ... ... ... ... ... | Sedgemoor Cottage, St. Auste |
| Matthews, Charles E., M.D. Dur. ... ... | 6 Hickman Road, Penarth. |
| Matthews, H. E. ... ... ... .. ... ... ... | The Mall Pharmacy, Clifton. |
| *Melsome, W. S., M.D. Cantab.... ... ... ... | 20 Circus, Bath. |

| | |
|---|---|
| Merck, E. | 16 Jewry Street, London, E.C. |
| Meredith, John, M.D. Ed. | Wellington, Somersetshire. |
| *Millard, W. J. Kelson, M.D. St. And. | 7 Bayshill Villas, Cheltenham. |
| Mitchell, Trafford, M.D. | Gorseinon, R.S.O., Glamorganshire. |
| *Mole, Harold F. | Royal Infirmary, Bristol. |
| Monckton, William | Portishead. |
| Morgan, Albert T., M.D. Brux. | 89 Chesterfield Road, St. Andrew's Park, Bristol. |
| Morgan, John | Langport, Somersetshire. |
| *Morgan, T. Whitworth S. | Pill. |
| *Morton, C. A. | 14 Vyvyan Terrace, Clifton. |
| *Morton, W. B., M.D. Lond. | Brislington House, Brislington. |
| Munden, Charles | Ilminster. |
| Muzumidar, Nogendranath, M.B. | Bhagalpore, Bengal, India. |
| *Myles, G. T. | 147 Whiteladies Road, Bristol. |

| | |
|---|---|
| *Nevill, W. N., M.D., B.Ch., B.A. Dub. | Leighton Villa, Southville, Bristol. |
| *Newman, Charles | 156 Cheltenham Road, Bristol. |
| *Newnham, W. H. C., M.B., M.A. Cantab. | Chandos Villa, Clifton. |
| Nicholson, T. D., M.D. Ed. | 2 Whiteladies Road, Clifton. |
| *Nield, Newman, M.B., B.Ch. Vict. | Richmond Hill, Clifton. |
| Norman, J. H. | Goodleigh, Winkleigh, Devonshire. |

| | |
|---|---|
| O'Brien, Cornelius | Devon House, Whitehall, Bristol. |
| Odell, William | Ferndale, Torquay. |
| *Ogilvy, Alexander, M.D., B.Ch., B.A. Dub. | Montrose House, Queen's Road, Clifton. |
| Openshaw, E. H. | Cheddar. |
| *Ormerod, H. Lawrence, M.B., B.Ch., B.A.O., R.U.I. | Burnley, Westbury-on-Trym |

| | |
|---|---|
| Page, G. Shepley | 78 Old Market Street, Bristol. |
| *Parker, George, M.D., M.A. Cantab. | 14 Pembroke Road, Clifton. |
| Parry, J. H. | 199 Cheltenham Road, Bristo |
| Parsons, Francis J. C. | Ivy House, Bridgwater. |
| *Parsons, H. F. | The Heath, Stoke Bishop. |
| Paterson, Donald Rose, M.D., C.M. Ed. | 18 Windsor Place, Cardiff. |
| Peake, Arthur W. | 34 Gloucester Road, Bishopston, Bristol. |
| Peake, G. Arthur | Rodney Place, Cheltenham. |
| *Pearse, E. M. | 51 Claredon Road, Redland, Bristol. |
| Peck, Major, R.A.M.C. | United Service Club, Calcutta. |
| *Perry, W. A. | 13 Dowry Square, Bristol. |
| *Phillips, C. M., M.D. Brux. | Ravenslea, Kensington Hill, Brislington. |
| Phillips, Edward Picton | High Street, Haverfordwest. |
| *Pickering, C. F. | 13 Berkeley Square, Bristol. |
| Plaister, W. H. | High Road, Tottenham. |
| Pollard, Reginald, M.B. Dur. | 8 Higher Terrace, Torquay. |
| *Pountney, W. E., M.D., C.M. Ed. | 33a Whiteladies Road, Bristol. |
| Powell, J. J., M.D. Lond. | 2 Portland Square, Bristol. |

*Powell, L. W. ... ... ... ... ... ... ... ... ... Two Mile Hill Road, Kingswood, Bristol.

Powell, William, M.B. Lond. ... ... ... ... Hill Garden, Torquay.

*Prichard, Arthur W. ... ... ... ... ... ... 6 Rodney Place, Clifton.

*Pringle, G. L. K., M.D. Edin. ... ... ... ... Bridgwater.

*Prowse, Arthur B., M.D. Lond. ... ... ... 5 Lansdown Place, Clifton.

Pruen, S. T., M.D. Dur. ... ... ... ... ... Sherborne Lodge, Cheltenham.

Purdy, J. R., M.D. Aber. ... ... ... ... ... Lower Hutt, New Zealand.

Randolph, Charles ... ... ... ... ... ... ... St. Michael's, Milverton, Somersetshire.
*Rattray, J. M., M.D., C.M., M.A. Aberd. ... Frome.
*Rayner, D. C. ... ... ... ... ... ... ... ... ... Oriel Villa, Cotham Road, Bristol.
Redwood, T. Hall, M.D., C.M., M.A.Dur. ... The Lawn, Rhymney, Monmouthshire.
Rendall, John ... ... ... ... ... ... ... ... Forestside, Lymington.
Riddell, Andrew W. ... ... ... ... ... ... ... New Brighton, Cheshire.
Roberts, Frederick T., M.D., B.Sc. Lond. 102 Harley Street, London, W.
Roderick, Sydney J., M.B., C.M. Ed. ... ... Llanelly.
Rodgers, John W., M.B., C.M. Ed. ... ... Redfield, Gloucestershire.
*Rogers, B. M. H., M.D., B.Ch., B.A. Oxon. ... 11 York Place, Clifton.
*Rose, F. H. ... ... ... ... ... ... ... ... ... Westfield Park, Clifton.
*Rossiter, George F., M.B. Lond. ... ... Cairo Lodge, Weston-super-Mare.
Roué, W. Barrett, M.D., M.S. Dur. ... ... 2 Buckingham Place, Clifton.
Routh, R. Henry F. ... ... ... ... ... ... ... Bridgwater.
*Roxburgh, Robert, M.B. Ed. ... ... ... 1 Victoria Buildings, Weston-super-Mare.
*Rudge, C. King ... ... ... ... ... ... ... ... 145 Whiteladies Road, Bristol.
*Rudge, H. T. ... ... ... ... ... ... ... ... ... 5 Colston Parade, Bristol.
Ruffer, M. Armand, M.D., B.S., M.B. Oxon. Alexandria, Egypt.
Rutherford, H. T., M.A., M.D. Camb. ... ... Taunton.

Scott, Richard J. H. ... ... ... ... ... ... ... 28 Circus, Bath.
Searle, George C. ... ... ... ... ... ... ... Brixham, Devonshire.
Searle, Richard Burford... ... ... .. ... Bexley, Dartmouth.
*Semple, W. R. M., M.B., C.M. Glasg. ... ... Beltrees, Yeovil, Somersetshire.
Seward, W. J., M.B. Lond. ... ... ... ... Asylum, Colney Hatch, London, N.
*Shaw, J. E., M.B., C.M. Ed. ... ... ... ... 23 Caledonia Place, Clifton.
Shaw, Jesse, M.D.... ... ... ... ... ... ... Fort Beaufort, Cape Colony.
Shaw-Mackenzie, John A., M.D. Lond. ... 31 Grosvenor Street, Grosvenor Square, W.
Sheen, A., M.D. St. And. ... ... ... ... ... 23 Newport Road, Cardiff.
*Sheppard, E. J. .. ... ... ... .. ... ... 8 Richmond Hill, Clifton.
*Shoolbred, W. A. ... ... ... ... ... ... ... ... St. Ann's, Chepstow.
Skelton, Henry, M.D. St. And. ... ... ... Downend, Gloucestershire.
*Skerritt, E. Markham, M.D., B.S., B.A. Lond. Richmond Hill, Clifton.
*Smith, G. Munro ... ... ... ... ... ... ... 18 Apsley Road, Clifton.
Smith, Rev. Reginald, M.A. Cantab. ... ... The Vicarage, Walpole St. Andrew, Wisbech, Norfolk.
*Smith, R. Shingleton, M.D., B.Sc. Lond. ... Clifton Park, Clifton.
Smith, W. Alexander, M.B., M.A. Oxon. ... Newport, Essex.
*Smith, W. A. L., M.A., M.B., B.C. Cantab. ... 8 Chamberlain Street, Wells.
Smyth, H. J. ... ... ... ... ... ... ... ... New Road, South Molton.
Smyth, J. C. ... ... ... ... ... ... ... ... ... Wells Road, Glastonbury.
Society, Cardiff Medical (*Hon. Lib.*, Queen Street, Cardiff).
Society, Manchester Medical ... ... ... Medical Society's Library, The Owens College, Manchester.
Society, Plymouth Medical (*Hon. Lib.*, J. Elliott Square).
Solly, Reginald V., M.B., B.S. Lond. ... ... 40 Southernhay, Exeter.
Solly, S. E. ... ... ... ... ... ... ... ... ... Colorado Springs, Colorado, U. S. A.

### LIST OF SUBSCRIBERS.

| | |
|---|---|
| Somer, James ... ... ... ... ... ... ... ... ... ... | Broad Clyst. |
| *Stack, E. H. E., B.A., M.B., B.C. Cantab. ... | Royal Infirmary, Bristol. |
| Stamp, W. D. ... ... ... ... ... ... ... ... ... | Ridgeway House, Plympton. |
| Statham, R. Whiteside ... ... ... ... ... ... | Cheddar, Somersetshire. |
| *Steele, Charles, M.D. Dur. ... ... ... ... | 17 Richmond Hill, Clifton. |
| *Steele, W. H., Lt.-Col., M.D. Dub. ... ... | 18 Mortimer Road, Clifton. |
| Steele-Perkins, E. B. ... ... ... ... ... ... ... | 3 Wilton Place, St. James', Exeter. |
| *Stephenson, J. B., M.B., B.S. Dur. ... ... | 19 Park Row, Bristol. |
| Stevens, W. H. ... ... ... ... ... ... ... ... | Zetland Road, Bristol. |
| *Stewart, J., B.A., R.U.I. ... ... ... ... ... | Dunmurry, Sneyd Park, Bristol. |
| *Stock, W. S. V., M.B., B.S. Lond. ... ... ... | 11 Elgin Park, Redland. |
| Stoddart, F. Wallis ... ... ... ... ... ... ... | Southey House, College Green, Bristol. |
| *Swain, James, M.D., M.S. Lond. ... ... ... | 4 Victoria Square, Clifton. |
| *Swayne, J. G., M.D. Lond. ... ... ... ... ... | 74 Pembroke Road, Clifton. |
| *Swayne, W. Carless, M.D. Lond. ... ... ... | St. Paul's Road, Clifton. |
| *Symes, J. O., M.D. Lond. ... ... ... ... ... | 11 Richmond Hill, Clifton. |
| Symonds, H. P. ... ... ... ... ... ... ... ... | 35 Beaumont Street, Oxford. |
| Symons, John ... ... ... ... ... ... ... ... ... | Buriton House, Penzance. |
| *Symons, W. H., M.D. Brux.... ... ... ... ... | Guildhall, Bath. |
| | |
| Taylor, C. Bell, M.D. Edin. ... ... ... ... | 9 Park Row, Nottingham. |
| *Taylor, James ... ... ... ... ... ... ... ... ... | 36 Alma Road, Clifton. |
| *Temple, G. H., M.B., C.M. Ed. ... ... ... | Weston-super-Mare. |
| Thomas, A. Garrod, M.D., C.M. Ed. ... ... | Clytha Park, Newport, Mon. |
| Thomas, J. Lynn ... ... ... ... ... ... ... ... | 28 Charles Street, Cardiff. |
| Thomas, Raglan, M.D. Lond. ... ... ... ... | 13 West Southernhay, Exeter. |
| Thomson, Eustace B., M.D., C.M. Glasg. ... | Albany Place, Plymouth. |
| *Tivy, W. J.... ... ... ... ... ... ... ... ... ... | 8 Lansdown Place, Clifton. |
| Tosswill, Louis H., M.B., B.A. Cantab.... ... | 1 West Southernhay, Exeter. |
| Trevithick, Edgar, M.A., M.D. Cantab. ... | 24 Promenade, Cheltenham. |
| Tyley, R. P., M.D. St. And.... ... ... ... ... | Wedmore. |
| | |
| Vachell, Herbert R., M.D., C.M. Aberd. ... | 18 Newport Road, Cardiff. |
| Vereker, R. H. ... ... ... ... ... ... ... ... | Eastleigh, Curry Rivel, Somerset |
| | |
| Wade, Charles H., M.A. Oxon. ... ... ... | Greenway, Cockington, Torquay. |
| *Waldo, Henry, M.D., C.M. Aberd. ... ... ... | 19 Pembroke Road, Clifton. |
| *Walker, Cyril H., M.B., B.A. Cantab. ... ... | 16 Pembroke Road, Clifton. |
| Wallace, Rev. Canon, M.A. Oxon. ... ... ... | 3 Harley Place, Clifton. |
| Wallace, J., M.B. ... ... ... ... ... ... ... ... | 112 York Road, Bedminster |
| *Wallace, John ... ... ... ... ... ... ... ... | 1 Oriel Terrace, Weston-super-Mare |
| Walsh, Leslie H., M.B., B.S. Durh. ... ... | 21 Gay Street, Bath. |
| Ward, J. L. W.... ... ... ... ... ... ... ... ... | Victoria Street, Merthyr Tydvi |
| *Wathen, J. Hancocke ... ... ... ... ... ... | 16 York Place, Clifton. |
| Watters, George T. B., M.D., C.M. Ed. ... | Stonehouse, Gloucestershire. |
| Weatherly, Lionel A., M.D., C.M. Aberd. | Bailbrook House, Bath. |
| Webb, J. B. ... ... ... ... ... ... ... ... ... ... | 10 Upper Barton Hill Road, Barton Hill, Bristol. |
| Webber, William W. ... ... ... ... ... ... ... | Crewkerne. |
| Whitby, C. J., B.A., M.D. Cantab. ... ... ... | Hillgrove-over-Wells, Somerset. |
| Wickham, W. ... ... ... ... ... ... ... ... ... | Hill House, Tetbury. |
| Wigan, C. A., M.D. Dur. ... ... ... ... ... | Portishead. |
| Wigmore, J., M.D. Roy. Univ. I. ... ... ... | 20 Green Park, Bath. |
| Wilcox, Henry, M.B. Aberd. ... ... ... ... | Fleet, Hampshire. |
| *Wilding, James, M.D. Dur. ... ... ... ... | 1 Apsley Villas, Kingsdown Parade, Bristol |

| | |
|---|---|
| Wilkin, Griffith C. ... ... ... ... ... ... | West Coker, Somerset. |
| Willcox, R. Lewis ... ... ... ... ... ... | Warminster. |
| Willett, George G. D. ... ... ... ... ... | Keynsham, Somersetshire. |
| *Williams, E. C., M.B., B.C., B.A. Cantab. ... | 9 Whatley Road, Clifton. |
| Williams, H. Egerton ... ... ... ... ... | The Mount, Newport, Mon. |
| Williams, John, M.D. ... ... ... ... ... ... | 20 Windsor Place, Cardiff. |
| *Williams, Lionel H. ... ... ... ... ... ... | Thornbury, Gloucestershire. |
| *Williams, P. Watson, M.D. Lond.... ... ... | 1 Victoria Square, Clifton. |
| Williams, Peter ... ... ... ... ... ... ... | Ferryside, Carmarthenshire. |
| *Williams, W. Roger ... ... ... ... ... ... | Beaufort House, Clifton Down, Clifton. |
| Willis, W. M.... ... ... ... ... ... ... ... | 24 Park Street, Nottingham. |
| *Wills, W. K., M.B., B.C. Camb. ... ... ... | 59 Apsley Road, Clifton. |
| *Windsor-Aubrey, H. W. ... ... ... ... ... | Coronation Road, Bristol. |
| *Wintle, Colston ... ... ... ... ... ... ... | 24 Gloucester Road, Bristol. |
| *Wohlmann, A. S., M.D., B.S. Lond. ... ... | 9 Gay Street, Bath. |
| Wood, C. R., M.A., M.D. Durh. ... ... ... ... | Bridge House, Frome. |
| Woodhead, G. Sims, M.D., C.M. Ed. ... ... | Pathological Laboratory, New Museum, Cambridge. |
| Woollcombe, Walter L. ... ... ... ... ... | 16 The Crescent, Plymouth. |

| | |
|---|---|
| *Young, J., M.D., C.M. Glasg. ... ... ... ... | Clouds Hill House, St. George, Bristol. |

### Local Medical Notes.

### BRISTOL.

Queen Victoria Jubilee Convalescent Home.—From the first report of this useful institution we obtain the following particulars:—

From the 30th of November, 1899, to the 31st December, 1900, the date at which the accounts close, 987 patients have been admitted. In the first ten months men and women were admitted in almost equal numbers (336 men, 351 women), in the last three months the women have exceeded the men by one-half (123 men, 187 women).

The average stay of each patient in the Home was 20 days. The patients have come from all sorts of employments, and a perusal of the registers of cases would convey a sense of the gravity of the illnesses and injuries from which the patients were recovering, and of the help that the Home has supplied to nearly a thousand sufferers.

Thanks to the home feeling the Matron by her kindliness diffuses, to the care the patients receive from the Medical Officer and the nurses, to the good feeding, and to the rest and the fine air, almost all who entered the Home have left it greatly improved in health, a large proportion quite cured.

Two deaths have occurred during the year.

On a review of the year's experience, the Governors consider that a building better suited to the purpose of the Home, or a position more desirable in every way, could not have been found than the house and grounds so generously given by the President, Sir Edward P. Wills, K.C.B.

The Governors, being most anxious to avoid incurring debt, felt that they could not allow to the Infirmary and Hospital more than 21 beds each, free of payment, out of the 30 originally allotted to each of them.

As it turned out, with the assistance of a cheque for £100, received on Christmas Day, as a present from the treasurer, the year closed with £107 15s. 9d. in hand.

Although the income for the current year will slightly exceed that of last year, which amounted to £2,155 15s. 4d., it seems as if it would not be possible without impairing the comfort of the patients, to provide for the 65 beds originally allotted to the medical institutions—still less to admit cases free of charge to the 15 beds reserved for patients not coming from those institutions.

In these circumstances, the treasurer, Mr. P. H. Vaughan, has most generously proposed to give a further sum to the Home of £10,000, on the condition that others will find an additional sum of £20,000 before the 31st of March, in order that the total number of beds allotted to the medical institutions may all be available for their use, and also that the whole of the remaining 15 beds may be free to suitable patients who have not been admitted from the institutions.

University College.—Dr. Newman Neild has been appointed Demonstrator of Physiology in the College. The following appointments have been gained by former students of the Bristol Medical School:—Mr. E. G. Bunbury, M.R.C.S., L.R.C.P., Medical Officer to the Clifton Dispensary; Mr. H. S. Jenkins, M.R.C.S., L.R.C.P., Junior House-Surgeon at the North London Hospital for Consumption; Mr. Gerald Grace, M.R.C.S., L.R.C.P., Surgeon to the Middleburgh District of the Transvaal. Mr. T. H. Parsons, F.R.C.S., has been awarded the British Medical Association Research Scholarship. At the recent Final M.B. Examination of the University of London Messrs. M. H. Phillips and W. Smith were successful, the former obtaining Honours in Obstetric Medicine. Mr. S. V. Stock obtained the B.S. degree. Mr. C. A. Moore was successful in the Intermediate Examination for M.B. Mr. J. H. Parsons, M.B., B.Sc. Lond., was successful in the Final, and Mr. A. R. Short, B.Sc. Lond., in the Primary F.R.C.S. Examinations.

Health of Bristol.—At the meeting of the Bristol Health Committee, held on January 8th, Dr. D. S. Davies, the Medical Officer of Health, reported that 8,972 births were registered during 1900, giving a birth-rate of 27.8 per 1,000, compared with 29 per 1,000 for 1899. This is the lowest birth-rate which Dr. Davies has recorded in Bristol. The number of deaths were 5,397, equal to a rate of 16.6 per 1,000, as against 18.2 per 1,000 in 1899. The infantile mortality under one year per 1,000 births was 182, which is the lowest recorded for the past ten years. The deaths registered from the principal zymotic diseases were equivalent to a rate of 1.8 per 1,000, the same as recorded in the previous year.

Medical Officers at Fever Hospitals.—At the meeting of the Bristol Town Council, held on January 2nd, the Sanitary Committee reported that although they had advertised for a Resident Medical Officer for the Ham Green Fever Hospital at a salary of £100 per annum, they were unable to obtain any suitable candidate. The Committee suggested that the salary be increased to £150 a year, and the Town Council eventually decided to agree to this proposal, an amendment to increase the salary to £200 per annum being defeated by 38 votes to 30.

Vaccination Grant.—Mr. G. Shepley Page has been awarded a grant by the Local Government Board for successful vaccination.

Bristol Dispensary.—The annual report for 1900 states that 10,060 patients were treated during the year, and that medical assistance was also given in 24 difficult midwifery cases. The committee add that the branch of the Dispensary at Bedminster is much appreciated, and that the number of patients attended is larger than in the preceding year. The financial statement shows that the number of subscriptions has declined during the past year.

### The Library of the Bristol Medico-Chirurgical Society.

*The following donations have been received since the publication of the List in December:*

*February 28th, 1901.*

| | |
|---|---|
| Sir William Broadbent, Bart. ... ... ... ... ... | 1 volume. |
| R. Caton, M.D. (1) ... ... ... ... ... ... ... ... ... | 1 ,, |
| Theodore Fisher, M.D. (2) ... ... ... ... ... ... | 1 ,, |
| L. M. Griffiths (3) ... ... ... ... ... ... ... ... | 3 volumes. |
| The Laryngological Society of London (4) ... ... | 1 volume. |
| The Otological Society of the United Kingdom (5) | 1 ,, |
| The Pathological Society of London (6) ... ... | 1 ,, |
| The Royal College of Physicians of London (7) | 2 volumes. |

The Surgeon-General, United States Army (8) ... 1 volume.

James Taylor ... ... ... ... ... ... ... ... ... 1 „

John Wright & Co. (9) ... ... ... ... ... ... ... 1 „

Unframed pictures have been presented by Dr. Christian Fenger, and Dr. T. Fisher. Unbound periodicals have been received from Dr. J. Michell Clarke and from the Medical Library of McGill University.

### THIRTY-NINTH LIST OF BOOKS.

The titles of books mentioned in previous lists are not repeated.

The figures in brackets refer to the figures after the names of the donors, and show by whom the volumes were presented. The books to which no such figures are attached have either been bought from the Library Fund or received through the *Journal*.

**Abrams, A.** ... ... *Transactions of the Antiseptic Club* ... ... ... ... ... 1900
**Babes, A.-V. Cornil et V.** *Les Bactéries* ... ... ... ... ... ... ... 1885
**Beck, C.** ... ... *Fractures* ... ... ... ... ... ... ... ... ... ... ... 1900
*Bibliotheca Meadiana* ... ... ... ... ... ... ... ... ... ... ... ... [1754]
**Bichat, X.** ... ... *Anatomie générale.* Ire part., Ier fasc. [Repr. of 1801 ed.] ... ... ... ... ... ... ... ... ... 1900
**Böhm and M. von Davidoff, A. A.** *A Text-Book of Histology* (Tr. from 2nd German edition by H. H. Cushing. Ed. by G. C. Huber) ... ... ... ... ... ... ... 1900
**Cantlie, J.** ... ... *Plague* ... ... ... ... ... ... ... ... ... ... ... 1900
**Carpenter, G.** ... *The Syphilis of Children in Every-day Practice* ... ... 1901
*Catalogue (Index) of the Library of the Surgeon-General's Office, United States Army.* 2nd Ser., Vol. V.... ... ... (8) 1900
**Caton, R.** ... ... *The Prevention of Valvular Disease of the Heart*... (1) 1900
**Celli, A.** ... ... *Malaria* (Tr. by J. J. Eyre) ... ... ... ... ... ... 1900
**Cheadle, W. B.** ... *Some Cirrhoses of the Liver* ... ... ... ... ... ... 1900
**Cornil et V. Babes, A.-V.** *Les Bactéries* ... ... ... ... ... ... ... 1885
*Cornwall as a Winter Resort* ... ... ... ... ... ... ... ... ... (3) 1900
**Cotgreave, A.** ... *A Contents-Subject Index* ... ... ... ... ... ... 1900
**Curtis, H. J.** ... *Practical Bacteriology* ... ... ... ... ... ... ... 1900
**Davidoff, A. A. Böhm and M. von.** *A Text-Book of Histology* (Tr. from 2nd German edition by H. H. Cushing. Ed. by G. C. Huber) ... ... ... ... ... ... ... 1900
"*Diarrhœa Deaths,*" *Certification and Classification of* ... ... ... ... ... 1900
**Easterbrook, C. C.** *Organo-Therapeutics in Mental Diseases* ... ... ... 1900
**Ellis, H.** ... ... *Studies in the Psychology of Sex* ... ... ... ... ... 1901
**Firth, J. L. Notter and R. H.** *The Theory and Practice of Hygiene.* 2nd Ed. by J. L. Notter and W. H. Horrocks ... 1900
**Gardner, H. B.** ... *The Asphyxial Factor in Anæsthesia* ... ... ... ... 1901
**Grandin and G. W. Jarman, E. H.** *Practical Obstetrics* ... ... 3rd Ed. 1900
**Hall and H. Tilley, F. de H.** *Diseases of the Nose and Throat.* 2nd Ed. 1901
**Hawkins, C.** ... *The Physical Examination and Development of School Boys* ... ... ... ... ... ... ... ... ... ... ... 1900
**Heisler, J. C.** ... *A Text-Book of Embryology* ... ... ... ... ... ... 1899
**Herman, G. E.** ... *Difficult Labour* ... ... ... ... ... ... [3rd] Ed. 1901
**Hovell, T. M.** ... *Diseases of the Ear and Naso-Pharynx* ... 2nd Ed. 1901
**Hunter, W.** ... *Oral Sepsis* ... ... ... ... ... ... ... ... ... ... 1901

**Jackson, E.** ... *Diseases of the Eye* ... 1900
**Jarman, E. H. Grandin and G. W.** *Practical Obstetrics* ... 3rd Ed. 1900
**Kelynack and W. Kirkby, T. N.** *Arsenical Poisoning in Beer Drinkers* ... 1901
**Kenwood, L. Parkes and H.** *Hygiene and Public Health* ... 1901
**Kirkby, T. N. Kelynack and W.** *Arsenical Poisoning in Beer Drinkers* ... 1901
**Klemperer, E. Levy and F.** *Clinical Bacteriology* (Tr. by A. A. Eshner).. 1900
**Lake, R.** ... *Laryngeal Phthisis* ... 1901
**Lawrie, E.** ... *Chloroform* ... 1901
**Levy and F. Klemperer, E.** *Clinical Bacteriology* (Tr. by A. A. Eshner).. 1900
**Macindoe, A.** ... *Sidmouth as a Health Resort* ... 1900
**Müller, J.** ... *Elements of Physiology* (Tr. by W. Baly). 2 vols. (3) 1838-42
**Murray, G. R.** ... *Diseases of the Thyroid Gland.* Part I. ... 1900
**Notter and R. H. Firth, J. L.** *The Theory and Practice of Hygiene.* 2nd Ed. by J. L. Notter and W. H. Horrocks ... 1900
**Ogden, J. B.** ... *Clinical Examinations of the Urine* ... 1900
**Oliver, G.** ... *Blood and Blood-Pressure* ... 1901
**Owen, [E.]** ... *Observations on the Earths, Rocks, Stones, and Minerals for some miles about Bristol* ... 1754
**Parkes and H. Kenwood, L.** *Hygiene and Public Health* ... 1901
**Payne, J. F.** ... *Thomas Sydenham* ... 1900
*Royal College of Physicians of London, List of the Portraits and other Works of Art in the* ... (7) 1900
,, ,, *Catalogue of Accessions to the Library of the, during* 1900 ... (7) 1900
**Senn, N.** ... *The Pathology and Surgical Treatment of Tumors.* 2nd Ed. 1900
**Sheild, A. M.** ... *Nasal Obstruction* ... 1900
**Shoemaker, J. V.**.. *Materia Medica and Therapeutics* ... 5th Ed. 1900
**Stengel, A.** ... *A Text-Book of Pathology* ... 3rd Ed. 1900
**Sutherland, G. A.** *Cyclic Albuminuria* ... 1900
**Tilley, F. de H. Hall and H.** *Diseases of the Nose and Throat* ... 2nd Ed. 1901
**Tunstall, F. J. Warwick and A. C.** *First Aid* ... 1901
**Venner, T.** ... *Via Recta ad Vitam Longam* ... (2) 1638
**Warwick and A. C. Tunstall, F. J.** *First Aid* ... 1901
**Williams, W. R.** . *Uterine Tumours* ... 1901
**Wise, T.** ... *How to Avoid Tubercle* ... 3rd Ed. 1900

### TRANSACTIONS, REPORTS, JOURNALS, &c.

Association of American Physicians, Transactions of the Vol. XV. 1900

Birmingham Medical Review, The ... Vols. XLVII., XLVIII. 1900

Bookseller, The ... 1900

Boston Medical and Surgical Journal, The... ...Vol. CXLIII. 1900

Bristol Directory ... (9) 1901

British Almanac, The ... 1901

British Medical Journal, The... ... Vol. II. for 1900

Centralblatt für Chirurgie ... 1900

Centralblatt für Gynäkologie... ... 1900

Clinical Society's Transactions ... Vol. XXXIII. 1900

Columbia University, Studies from the Department of Pathology of the College of Physicians and Surgeons ... Vol. VII. 1899-1900

Écho médical du Nord, L' ... ... ... ... ... ... ... ... ... ... 1900
Edinburgh Medical Journal, The ... ... ... ... ... N.S., Vol. VIII. 1900
English Catalogue, The ... ... ... ... ... ... ... ... ... ... 1890-97
Epidemiological Society of London, Transactions of the N.S., Vol. XIX. 1900
Gazette de Gynécologie ... ... ... ... ... ... ... ... Tome XV. 1900
Gazette des Hôpitaux ... ... ... ... ... ... ... ... ... ... ... 1900
Gazette des Hôpitaux de Toulouse ... ... ... ... ... ... ... ... 1900
Gazette hebdomadaire des Sciences médicales de Bordeaux Tome XXI. 1900
Gazette médicale de Paris ... ... ... ... ... 11e Sér., Tome III. 1900
Glasgow Medical Journal, The ... ... ... ... ... ... Vol. LIV. 1900
Hazell's Annual ... ... ... ... ... ... ... ... ... ... ... ... ... 1901
Hospital, The ... ... ... ... ... ... ... ... ... Vol. XXVIII. 1900
Hospitals—

Johns Hopkins Hospital Reports, The Vols. VII. No. 3, 1898, VIII. Nos. 3-9 1900

Middlesex Hospital Reports for 1898 ... ... ... ... ... ... 1899

St. Bartholomew's Hospital Reports ... ... Vol. XXXVI. 1901

Journal of Experimental Medicine, The ... ... ... ... ... Vol. IV. 1899
Journal of the Boston Society of Medical Sciences ... Vol. IV. 1899-1900
Lancet, The ... ... ... .. ... ... ... ... ... ... Vol. II. for 1900
Laryngological Society of London, Proceedings of the (4) Vol. VII. 1900
Library, The ... ... .. ... ... ... ... ... ... Vols. I., II. 1889-90
Medical Chronicle, The ... ... ... ... ... ... 3rd Ser., Vol. III. 1900
Medical Directory, The ... ... ... ... ... ... .. ... ... ... ... 1901
Medical Press and Circular, The ... ... ... ... ... [Vol. CXXI.] 1900
Mercy and Truth ... ... ... ... ... ... ... ... ... ... ... Vol. IV. 1900
Münchener medicinische Wochenschrift ... ... ... ... ... ... ... 1900
New Hampshire Medical Society, Transactions of the ... ... ... ... 1900
New York Medical Journal, The ... ... ... ... ... ... Vol. LXXII. 1900
Nord médical, Le ... ... ... ... ... ... ... ... ... Tome VI. 1900
Obstetrical Transactions ... ... ... ... ... ... ... ... Vol. XLI. 1900
Odontological Society of Great Britain, Transactions of the Vol. XXXII. 1900
Otological Society of the United Kingdom, Transactions of the (5) Vol. I. 1900
Pathological Society of London, Transactions of the ... (6) Vol. LI. 1900
Philadelphia Medical Journal, The ... ... ... ... ... ... Vol. VI. 1900
Polyclinic, The ... ... ... ... ... ... ... ... ... ... ... Vol. III. 1900
Practitioner, The ... ... ... ... ... ... ... ... ... Vol. LXV. 1900
Progressive Medicine ... ... ... ... ... ... ... Vols. III., IV. 1900-1
Public Health ... ... ... ... ... ... ... ... ... Vol. XII. 1899—1900
Quarterly Medical Journal, The ... ... ... ... ... Vol. VIII. 1899—1900
Retrospect of Medicine, Braithwaite's ... ... ... ... ... Vol. CXXII. 1901
Revue de Thérapeutique médico-chirurgicale ... ... ... ... ... ... 1900
Revue hebdomadaire de Laryngologie ... ... ... ... Tome XX., Vol. 2 1900
Royal Academy of Medicine in Ireland, Transactions of the Vol. XVIII. 1900
Sanitary Record, The ... ... ... ... ... ... ... ... ... Vol. XXVI. 1900
Scottish Medical and Surgical Journal, The ... ... ... Vol. VII. 1900
Whitaker's Almanack ... ... ... ... ... ... ... ... ... ... ... 1901
Who's Who ... ... ... ... ... ... ... ... ... ... ... ... ... ... 1901
Wiener klinische Wochenschrift ... ... ... ... ... ... ... ... ... 1900

